# Metabolic Reprogramming of T Cells by MSCs Rebalances Th17/Treg Axis to Attenuate Collagen‐Induced Arthritis

**DOI:** 10.1155/jimr/1862250

**Published:** 2026-06-08

**Authors:** Xiaoping Wang, Jingjing He, Qun Wang, Xue Liu, Haoming Yuan, Lu Jin, Meng Ding, Lin Yang, Shaoxin Cui, Fei Chang, Tong Xin, Hongtao Jin, Min Shi, Yongzhou Song, Wensen Pan, Aijing Liu

**Affiliations:** ^1^ Department of Rheumatology and Immunology, The Second Hospital of Hebei Medical University, Shijiazhuang, Hebei, China, hebmu.edu.cn; ^2^ Hebei International Joint Research Center on Rheumatic Diseases, The Second Hospital of Hebei Medical University, Shijiazhuang, Hebei, China, hebmu.edu.cn; ^3^ Department of Respiratory and Critical Care Medicine, The Second Hospital of Hebei Medical University, Shijiazhuang, Hebei, China, hebmu.edu.cn; ^4^ Department of Public Health, International College, Krirk University, No. 3 soi Ramintra 1, Ramintra Road, Anusaowaree, Bangkhen, Bangkok, 10220, Thailand, krirk.ac.th; ^5^ Department of Clinical Laboratory, The Second Hospital of Hebei Medical University, Shijiazhuang, Hebei, China, hebmu.edu.cn; ^6^ Hebei Key Laboratory of Laboratory Medicine, The Second Hospital of Hebei Medical University, Shijiazhuang, Hebei, China, hebmu.edu.cn; ^7^ Department of Orthopedics, The Second Hospital of Hebei Medical University, Shijiazhuang, Hebei, China, hebmu.edu.cn; ^8^ Hebei Research Center for Stem Cell Medical Translational Engineering, Hebei Medical University, Shijiazhuang, Hebei, China, hebmu.edu.cn

**Keywords:** collagen-induced arthritis, glycolysis, mesenchymal stromal cells, rheumatoid arthritis, T cell metabolism, Th17/Treg balance

## Abstract

**Background:**

Rheumatoid arthritis (RA) is a chronic autoimmune disorder characterized by dysregulated T cell responses and metabolic disturbances. Mesenchymal stromal cells (MSCs) have shown therapeutic promise, but their mechanisms, particularly concerning T cell metabolism, remain incompletely defined. This study investigated whether human umbilical cord‐derived MSCs (hUC‐MSCs) ameliorate collagen‐induced arthritis (CIA) by modulating T cell metabolism and differentiation.

**Methods:**

CIA was induced in DBA/1 mice. Animals received PBS or hUC‐MSCs on day 28. Arthritis index (AI), joint histology, serum cytokines (TNF‐α, IL‐6, IL‐17, and TGF‐β), and metabolites (lactate and pyruvate) were assessed. Splenic T cell transcription factors (*FOXP3*, *RORγt*, and *PU.1*) and glycolytic genes (*GLUT1*, *G6PD*, and *PFKFB3*) were analyzed by real‐time quantitative polymerase chain reaction (RT‐qPCR) and western blot. *In vitro*, human CD4^+^ T cells were cocultured with hUC‐MSCs under T‐helper 17 (Th17)‐polarizing conditions. T cell subsets, glycolytic metabolites, and gene/protein expression were evaluated by flow cytometry, colorimetric assays, RT‐qPCR, and western blot.

**Results:**

MSC treatment significantly attenuated arthritis severity, joint destruction, and splenomegaly in CIA mice. It reduced serum pro‐inflammatory cytokines and normalized elevated lactate and pyruvate levels. In the spleen, MSCs suppressed *RORγt* and *PU.1* while enhancing *FOXP3* expression, and downregulated *GLUT1* and *G6PD* mRNA. Positive correlations were found between glycolytic markers (*GLUT1* and *G6PD*) and pro‐inflammatory transcription factors (*RORγt* and *PU.1*), and between serum lactate and inflammatory cytokines. *In vitro*, hUC‐MSCs directly inhibited Th17 differentiation and promoted Treg generation in human CD4^+^ T cells. This metabolic reprogramming was functionally coupled to a shift in T cell differentiation: a suppression of pro‐inflammatory Th17 cells and a promotion of regulatory T (Treg) generation in human CD4^+^ T cells. This was accompanied by reduced lactate production and significant downregulation of *GLUT1*, *G6PD*, and *PFKFB3* at both mRNA and protein levels.

**Conclusions:**

hUC‐MSCs ameliorate CIA by restoring the Th17/Treg balance through metabolic reprogramming of T cells, specifically by suppressing glycolysis. This immunometabolic mechanism highlights the therapeutic potential of MSCs in RA.

## 1. Introduction

Rheumatoid arthritis (RA) is a chronic autoimmune disease characterized by persistent synovial inflammation, progressive joint destruction, and systemic complications [1]. The pathogenesis of RA involves a complex interplay of innate and adaptive immune responses, with dysregulated T‐cell activation playing a central role. In particular, an imbalance between pro‐inflammatory T‐helper 17 (Th17) cells and regulatory T (Treg) cells contributes significantly to synovitis and tissue damage [2]. Activated T cells in RA exhibit a metabolic shift toward glycolysis, which provides the energy and biosynthetic precursors required for their proliferation and cytokine production [3]. This immunometabolic reprogramming is increasingly recognized as a key driver of RA pathology and a potential therapeutic target [4, 5]. The development of RA is further complicated by the interaction of immune cells—such as dendritic cells, macrophages, T and B lymphocytes, and neutrophils—with osteoclasts that are excessively activated, leading to progressive destruction of cartilage and bone [6, 7]. Epidemiological data from China suggest that RA affects between 0.28% and 0.43% of the population, with a female‐to‐male‐ratio of approximately 4:1, most commonly affecting individuals between the ages of 25 and 55 [8]. In its early stages, symptoms include joint pain, swelling, and morning stiffness. As the disease advances, the progressive breakdown of bone and cartilage can cause severe joint dysfunction, which in some cases may lead to disability [9]. This condition can also reduce life expectancy by approximately 6–7 years [10].

The etiology of RA remains incompletely understood but involves genetic predisposition, infections, and environmental factors such as smoking. Dysregulated immune responses, particularly aberrant T cell activity, play a central role in RA pathogenesis. Imbalances in Th cell subsets, notably overactivation of Th1 and Th17 cells, contribute to synovial inflammation and joint damage [11]. These observations highlight the significant involvement of T cells in RA development and progression. T cells, particularly during their activation and differentiation, are heavily reliant on glucose as a primary energy source. When activated, T cells enhance glycolysis, a metabolic process that supplies the necessary energy for the production of inflammatory mediators [12]. Alterations in glucose metabolism within T cells have been strongly linked to RA pathogenesis, where disturbances in CD4^+^ T cell metabolism promote irregular proliferation and differentiation typical of RA [13].

Current treatments, including conventional disease‐modifying antirheumatic drugs (DMARDs) and biologic agents, often have limited efficacy or are associated with significant side effects [11], and many patients experience suboptimal response or adverse effects, underscoring the need for novel therapies. Mesenchymal stromal cells (MSCs) have emerged as a promising regenerative and immunomodulatory therapy for RA due to their ability to suppress excessive immune responses and promote tissue repair [14–16]. Preclinical and clinical studies have demonstrated that MSCs can attenuate joint inflammation, reduce cartilage erosion, and rebalance Th17/Treg ratios in experimental arthritis models [14, 17–20]. However, the mechanisms underlying these effects, particularly whether MSCs modulate T cell metabolism to restore immune homeostasis, remain incompletely understood [21].

Given the critical role of glucose metabolism in T‐cell differentiation and function, we hypothesized that MSCs exert their therapeutic effects in part by reprogramming glycolytic pathways in T cells, thereby shifting the Th17/Treg equilibrium. In this study, we employed a collagen‐induced arthritis (CIA) mouse model and an *in vitro* human T‐cell coculture system to investigate the effects of human umbilical cord‐derived MSCs (hUC‐MSCs) on T‐cell metabolism and differentiation. Our findings demonstrate that hUC‐MSCs alleviate arthritis severity, reduce pro‐inflammatory cytokines, and correct metabolic disturbances *in vivo* while directly suppressing glycolysis and promoting Treg generation in human CD4^+^ T cells *in vitro*. These results provide novel insights into the immunometabolic mechanisms of MSC‐based therapy and support their potential as a multifaceted treatment strategy for RA.

## 2. Materials and Methods

### 2.1. Experimental Animals

A total of 28 male DBA/1 mice (aged 6–7 weeks; 18–22 g) were obtained from Beijing Vital River Laboratory Animal Technology Co., Ltd. Mice were housed in a specific pathogen‐free (SPF) facility with a controlled environment, where temperature (20–22°C), humidity (50% ± 10%), and a 12‐h light/dark cycle were maintained. Food and water were provided *ad libitum*. After a 1‐week acclimatization period, the experiments commenced.

All protocols were approved by the Institutional Animal Care and Use Committee (IACUC) of the Second Hospital of Hebei Medical University (approval number: 2023‐AE‐164) and complied with the National Institutes of Health (NIH) Guide for the Care and Use of Laboratory Animals (8th edition, 2011).

### 2.2. MSC Culture

hUC‐MSCs (Qilu Cell Therapy Engineering Technology Co., Ltd., China; Catalog No. PUM‐A1−000082) were cultured in DMEM/F12 medium supplemented with 10% fetal bovine serum (FBS), 1% GlutaMAX, and 1% penicillin–streptomycin. Cells were maintained at 37°C in a 5% CO_2_‐humidified incubator, with media changes every 2–3 days. Passages 3–5 (P3–P5) were used for experiments.

### 2.3. CIA Model Establishment

CIA was induced in DBA/1 mice using a two‐phase protocol. Bovine type II collagen (CII, Chondrex, USA) and complete Freund’s adjuvant (CFA, Chondrex, USA) were emulsified and injected intradermally at the tail base (0.1 mL) under isoflurane anesthesia as a prime immunization. CII and incomplete Freund’s adjuvant (IFA, Chondrex, USA) were administered as a boost immunization 21 days later.

**Figure 1:**
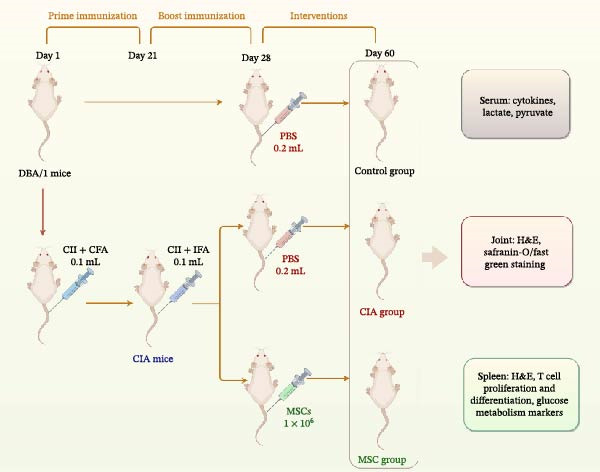
Diagram of mouse modeling and treatment. DBA/1 mice were immunized with bovine type II collagen (CII) and Freund’s adjuvant to establish the CIA model. On day 28, mice were randomized to receive either PBS or 1 × 10^6^ hUC‐MSCs via tail vein injection. Clinical arthritis index (AI) and paw thickness were monitored weekly until sacrifice on day 60.

On day 28 post‐immunization, mice with an arthritis index (AI) score exceeding 2 were randomized into CIA (*n* = 10, PBS injection) and MSC (*n* = 10, 1 × 10^6^ hUC‐MSCs in PBS) groups. A control group (*n* = 8) received 0.2 mL of PBS alone. Paw thickness and AI scores (0–16 scale) were recorded weekly (Figure 1).

### 2.4. Sample Collection

On day 60, blood was collected via cardiac puncture, and the serum was stored at −80°C. Mice were euthanized by cervical dislocation, followed by sterilization of the abdominal skin. The spleen, knee joints, and synovial tissues were collected. Some portions were rapidly frozen in liquid nitrogen and stored in 1.5 mL microcentrifuge tubes at −80°C, while the remaining tissues were fixed in 4% paraformaldehyde, decalcified using EDTA (Solarbio, China).

### 2.5. Spleen Index

The spleen was weighed and the spleen index (spleen weight/body weight × 100%) was calculated.

### 2.6. Hematoxylin and Eosin (H&E) Staining

Samples were fixed in 4% paraformaldehyde, paraffin‐embedded, and sectioned into 4‐µm slices. Following dewaxing, tissues were rehydrated through gradient ethanol solutions (Sinopharm, China). Nuclei were stain with hematoxylin (Solarbio, China) for 5 min, followed by 2 min eosin staining for cytoplasmic visualization. Sections were dehydrated in ascending alcohol concentrations, cleared with xylene (Sinopharm, China), and coverslipped. Histological changes (inflammatory responses and tissue architecture) were observed by light microscopy.

### 2.7. Safranin‐O/Fast Green Staining

Five‐micrometer joint sections were stained with safranin‐O and fast green (Solarbio, China) according to the manufacturer’s protocol. Safranin‐O‐stained proteoglycans appeared red, while fast green counterstained non‐cartilage tissues. Cartilage condition was evaluated microscopically, and proteoglycan depletion was scored (0–3), where 0 = no depletion and 3 = complete loss of safranin‐O staining.

### 2.8. Immunohistochemistry (IHC)

Cell proliferation in spleen tissues was assessed by IHC for Ki67. Paraffin‐embedded spleen tissues were dewaxed in xylene and hydration through gradient ethanol solutions. Antigen retrieval was achieved by heating the sections in citrate buffer (pH 6.0) at 95°C for 20 min. Endogenous peroxidase activity was blocked with 3% hydrogen peroxide for 10 min, followed by 30 min of pretreatment with 5% bovine serum albumin to reduce nonspecific binding. Sections were incubated overnight at 4°C with a Ki67 primary antibody (Servicebio, China), then probed with a biotin‐labeled secondary antibody for 1 h. Signal amplification was performed using streptavidin–HRP complex, and the immunoreactivity was visualized with diaminobenzidine. Proliferating cells were identified by positive Ki67 nuclear staining, and their distribution and density were evaluated under a light microscope.

### 2.9. Enzyme‐Linked Immunosorbent Assay (ELISA)

Cytokines levels (TNF‐α, IL‐6, IL‐17, and TGF‐β) were quantified using ELISA kits (ABclonal, China) according to the manufacturer’s instructions. Serum samples were diluted, and 100 μL was added to microplate wells, followed by 2 h incubation at 37°C. Plates were washed to remove unbound substances, and detection antibodies were applied for 1 h. Absorbance was measured at 450 nm using a microplate reader with triplicate measurements for each sample to ensure reliability.

### 2.10. Colorimetry

Lactate and pyruvate were measured using a colorimetric assay kit (Nanjingjiancheng, Jiangsu, China) per manufacturer’s protocols, and the ratio of lactate‐to‐pyruvate was analysized were quantified. Briefly, 50‐µL serum or culture supernatant was mixed with 100‐µL of assay reagent in 96‐well plates, incubated at 37°C for 30 min, and absorbance was measured at 540 nm using a microplate reader. Concentrations were calculated by referencing standard curves, with triplicate measurements for each sample.

### 2.11. Isolation and Purification of Human CD4^+^ T Cells

Human peripheral blood samples were collected from healthy donors. PBMCs were isolated by density gradient centrifugation using Ficoll‐PaquePLUS (Cytiva) according to the manufacturer’s instructions. Briefly, blood was diluted 1:1 with PBS, carefully layered over an equal volume of Ficoll, and centrifuged at 800 × *g* for 20 min at room temperature with low acceleration and deceleration. The PBMC layer was collected, washed three times with PBS, and resuspended in complete RPMI‐1640 medium (HyClone) supplemented with 10% heat‐inactivated FBS (HyClone) and 1% penicillin–streptomycin. CD4^+^ T cells were positively selected from PBMCs using anti‐human CD4 microbeads (Miltenyi Biotec, 130–045−101) and LS columns (Miltenyi Biotec) according to the manufacturer’s protocol. The purity of isolated CD4^+^ T cells was routinely >95%, as verified by flow cytometry.

### 2.12. *In Vitro* Coculture

Purified CD4^+^ T cells were seeded into six‐well plates at a density of 1 × 10^6^ cells per well. HUC‐MSCs were added at a ratio of 1:10 (MSC:T cell = 1:10). Cells were cultured in Th17‐polarizing medium (UA090019, Youai Bio) containing recombinant human IL‐6 (20 ng/mL), IL‐23 (10 ng/mL), TGF‐β (5 ng/mL), anti‐IFN‐γ (10 µg/mL), and anti‐IL‐4 (10 µg/mL), along with plate‐bound anti‐CD3 (1 µg/mL) and soluble anti‐CD28 (1 µg/mL). The coculture was maintained for 5 days at 37°C in a 5% CO_2-_humidified incubator, with medium replaced every 2–3 days. After 5 days of coculture, T cells were carefully collected by gentle pipetting to minimize contamination by adherent hUC‐MSCs. Cells were then processed for further analysis.

### 2.13. Flow Cytometry

Cells were incubated with fluorochrome‐conjugated anti‐human CD4 (FITC) and anti‐CD25 (PerCP) antibodies at 4°C for 30 min. For intracellular detection, cells were fixed, permeabilized and stained with anti‐Foxp3 (PE) and anti‐RORγt (PE‐Cy7) using a Foxp3/transcription factor staining buffer set (eBioscience). To assess IL‐17A production, cells were restimulated with PMA/ionomycin for 4–6 h in the presence of protein transport inhibitors prior to intracellular staining with anti‐IL‐17A (PE‐Cy7). The gating strategy (Figures S5E and S10F) sequentially identified lymphocytes (FSC/SSC), singlets (FSC‐A/FSC‐H), and CD4^+^ T cells. Th17 cells were defined as CD4^+^IL‐17 A^+^ or CD4^+^RORγt^+^, and Tregs as CD4^+^CD25^+^Foxp3^+^. All samples included appropriate isotype and single‐stain controls (Blank). Samples were analyzed on a BD FACSAria III flow cytometer, and data were processed using FlowJo software (v10.8.1). Th17 cells were identified as CD4^+^IL‐17 A^+^ or CD4^+^RORγt^+^, and Tregs as CD4^+^CD25^+^Foxp3^+^. A minimum of 10,000 lymphocyte‐gated events were acquired per sample.

### 2.14. Real‐Time Quantitative Polymerase Chain Reaction (RT‐qPCR)

Total RNA was isolated from mouse spleen tissues or cultured human CD4^+^ T cells using TRIzol reagent (Invitrogen, USA) following the manufacturer’s protocol. Reverse transcription was performed with the PrimeScript RT Reagent Kit (Takara Bio, Japan) to synthesize cDNA. RT‐qPCR was conducted on a StepOnePlus system (Roche LightCycler 480 system, Switzerland) using SYBR green master mix (Thermo Fisher Scientific, USA). Gene‐specific primers for *FOXP3*, *RORγt*, *PU.1*, *GLUT1*, *G6PD*, *PFKFB3*, and reference gene*GAPDH/β-actin* are listed in Table 1. All samples were analyzed in triplicate (technical replicates) from at least three independent experiments (biological replicates). Relative gene expression was calculated via the 2^−ΔΔCt^ method, with normalization to the reference gene as an internal control.

**Table 1:** Primer sequences of all RT‐qPCR reactions.

Gene	Direction	Sequence (5′–3′)
*Mus ROR-γt*	Forward primer	CCTGGGCTACCCTACTGAGGA
	Reverse primer	GCTTCTTGGACATTCGGCCA
*Mus PU.1*	Forward primer	AGAAGCTGATGGCTTGGAGC
	Reverse primer	TTTGTCCTTGTCCACCCACC
*Mus FOXP3*	Forward primer	GACCCCCTTTCACCTATGCC
	Reverse primer	TGAAGTAGGCGAACATGCGA
*Mus GLUT1*	Forward primer	TGTTCATTGGTTTGGGGCCT
	Reverse primer	CCGCAAAAACCTGAGAAGCG
*Mus G6PD*	Forward primer	GGAATCAGGGCCTAAGGTCA
	Reverse primer	AGAGAGACCAAAGCGTGGTG
*Mus PFKFB3*	Forward primer	TGTTCATTGGTTTGGGGCCT
	Reverse primer	CCGCAAAAACCTGAGAAGCG
Mus GADPH	Forward primer	CATCACTGCCACCCAGAAGACTG
	Reverse primer	ATGCCAGTGAGCTTCCCGTTCAG
*Mus β-actin*	Forward primer	GTACCCAGGCATTGCTGACA
	Reverse primer	AACGCAGCTCAGTAACAGTC
*Homo GLUT1*	Forward primer	ATGGGCTTCTCGAAACTGGG
	Reverse primer	CCGCAGTACACACCGATGAT
*Homo G6PD*	Forward primer	CTACCGCATCGACCACTACC
	Reverse primer	TGTTGTCCCGGTTCCAGATG
*Homo PFKFB3*	Forward primer	GTGCCTTAGCTGCCTTGAGA
	Reverse primer	GTGGCATCGAAAACCGCAAT
*Homo GADPH*	Forward primer	ACGGATTTGGTCGTATTGGG
	Reverse primer	GGGATCTCGCTCCTGGAAG

### 2.15. Western Blot

Total protein was extracted from mouse spleen tissues or human CD4^+^ T cells using RIPA lysis buffer supplemented with protease and phosphatase inhibitors. Protein concentration was determined by the BCA assay. Equal amounts of protein (20 µg per lane) were separated by SDS–PAGE and transferred onto PVDF membranes. After blocking with 5% non‐fat milk, membranes were incubated overnight at 4°C with primary antibodies against target proteins: for mouse spleen tissues—RORγt, FOXP3, PU.1, and IL‐9; for human CD4^+^ T cells—GLUT1, G6PD, and PFKFB3. β‐actin or GAPDH was used as loading control. Following incubation with an HRP‐conjugated secondary antibody, protein bands were visualized using enhanced chemiluminescence and quantified by densitometry. To ensure data transparency and reproducibility, the full‐length, uncropped images of the western blots for all protein targets are provided in the Supporting Information File S1.

### 2.16. Statistical Analysis

Data are presented as mean ± standard deviation (SD). Normality and homogeneity of variance were assessed using the Shapiro–Wilk and Levene’s tests, respectively. Group comparisons were performed using an unpaired two‐tailed Student’s *t*‐test (for two groups) or one‐way ANOVA followed by Tukey’s *post hoc* test (for multiple groups). For longitudinal arthritis scoring, repeated‐measures two‐way ANOVA with Bonferroni correction was applied. Nonparametric data were analyzed using the Mann–Whitney *U* test or Kruskal–Wallis test followed by Dunn’s *post hoc* test. Correlations were evaluated using Pearson or Spearman tests depending on data distribution. Statistical analyses were performed using SPSS (v. 25.0) and GraphPad Prism (v. 9.1.2). *p* < 0.05 were considered statistically significant.

## 3. Results

### 3.1. Amelioration of CIA General Condition by MSCs

**Figure 2:**
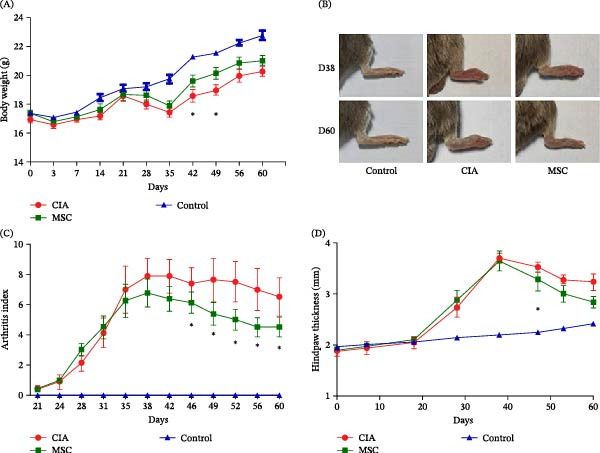
MSCs alleviate the general condition of CIA mice. (A) Mouse body weights were recorded from days 0 to 60 (control *n* = 4, CIA *n* = 10, and MSC *n* = 9). (B) Representative photographs captured at the end of the study show a marked reduction in joint swelling in mice treated with MSCs. (C) AI scores recorded from days 21 to 60 (control *n* = 4, MSC *n* = 8, and CIA *n* = 8). (D) Paw thickness was recorded from days 0 to 60 (control *n* = 4, CIA *n* = 8, and MSC *n* = 8). Date are presented as mean ± SD and were analyzed via one‐way ANOVA followed by Tukey’s *post hoc* tests.  ^∗^ *p* < 0.05 CIA vs. MSC.

CIA mice exhibited reduced activity, coarser fur, and impaired weight gain. MSC treatment restored body weight (*p* < 0.05, Figure 2A) and improved joint swelling (Figure 2B). AI scores peaked on day 38 and declined thereafter, with MSC‐treated mice showing significant reductions by day 45 (*p* < 0.05, Figure 2C). Paw thickness also decreased in MSC group (*p* < 0.05, Figure 2D).

### 3.2. Preservation of Joint and Spleen Architecture by MSCs

**Figure 3:**
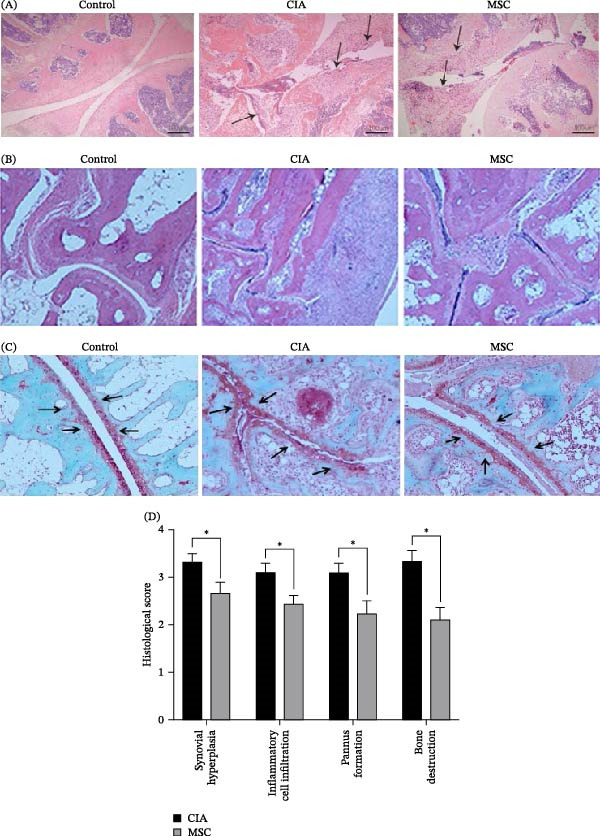
Effect of MSCs on the pathological structure of joints. (A) H&E staining of knee joint. (B) H&E staining of ankle joint. (C) Safranin‐O/fast green staining of knee joint. (D) Pathological score (CIA *n* = 9 and MSC *n* = 9). Date are presented as mean ± SD and were analyzed via unpaired two‐tailed Student’s *t*‐test.  ^∗^ *p* < 0.05 CIA vs. MSC.

Control group joints exhibited preserved tissue architecture without synovial proliferation or bone degradation. Cartilage displayed uniform matrix integrity, normal thickness, and distinct tidemark formation (Figure 3A–C). In contrast, CIA‐induced joints showed significant structural disruption, featuring synovial invasion, cartilage matrix reduction, irregular tidemark, and bone damage. MSC treatment substantially restored joint morphology, demonstrating improved cartilage preservation and bone integrity (*p* < 0.05 vs. CIA group). The CIA group developed severe synovitis characterized by pannus formation, inflammatory cell infiltration, and joint destruction (Figure 3D). MSC intervention significantly mitigated these pathological changes, reducing synovial hyperplasia (*p* < 0.05) and preserving joint surfaces.

**Figure 4:**
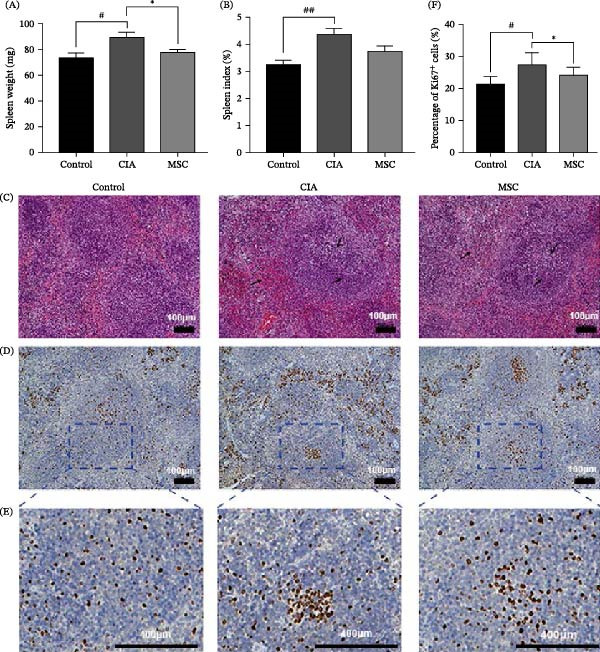
Effect of MSCs on the pathological structure of the spleen. (A) Spleen weights recorded at the end of the study (control *n* = 4, CIA *n* = 10, and MSC *n* = 10). (B) Spleen index (control *n* = 4, CIA *n* = 10, and MSC *n* = 10). (C) Representative H&E staining images (scale bars, 100 μm). (D) Representative Ki67 staining images (scale bars, 100 μm). (E) Representative zoom‐in images of Ki67 staining (scale bars, 400 μm) and (F) quantitative analysis. Data are presented as mean ± SD and were analyzed via one‐way ANOVA followed by Tukey’s *post hoc* tests. ^#^ *p* < 0.05 Control vs. CIA, ^##^ *p* < 0.01 control vs. CIA, and  ^∗^ *p* < 0.05 CIA vs. MSC.

CIA induction significantly increased spleen weight (*p* < 0.05) and spleen index (*p* < 0.01) vs. controls. MSC treatment normalized spleen weight (*p* < 0.05, Figure 4A) but showed non‐significant reduction in spleen index (*p* > 0.05, Figure 4B). Histologically, CIA spleens exhibited germinal centers expansion, red pulp congestion, and white pulp hyperplasia‐all ameliorated by MSC administration (Figure 4C). IHC quantification revealed elevated Ki67^+^ cells in CIA spleens, which MSC treatment significantly reduced (*p* < 0.05, Figure 4D–F).

### 3.3. Modulation of Transcriptional and Metabolic Profiles by MSCs

**Figure 5:**
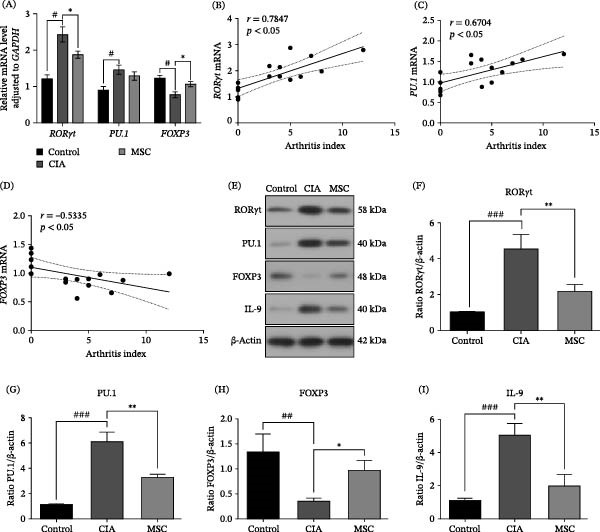
Effect of MSCs on T lymphocyte differentiation in the spleen. (A) Relative mRNA levels of *RORγt*, *PU.1*, and *FOXP3* in spleen. (B) Correlations between AI and *FOXP3* mRNA. (C) Correlations between AI and *RORγt* mRNA. (D) Correlations between AI and *PU.1* mRNA. (E) Western blot analysis of RORγt (F), PU.1 (G), FOXP3 (H), and IL‐9 (I) expression in mouse spleen after cryogenic grinding. All uncropped raw western Blot images are provided in the “Original Blot Data” PDF file. Data represents mean ± SD from at least three independent experiments (Control *n* = 3, CIA *n* = 3, and MSC *n* = 3). Date were analyzed via one‐way ANOVA followed by Tukey’s *post hoc* tests. Correlations were evaluated using Pearson or Spearman tests. ^#^ *p* < 0.05 Control vs. CIA, ^##^ *p* < 0.01 control vs. CIA, ^###^ *p* < 0.0001 control vs. CIA,  ^∗^ *p* < 0.05 CIA vs. MSC, and  ^∗∗^ *p* < 0.01 CIA vs. MSC.

Key transcription factors in spleen tissue were analyzed using RT‐qPCR and western blot. Compared to controls, CIA mice exhibited significantly elevated levels of *RORγt* (Th17‐associated) and *PU.1* (Th9‐associated), alongside reduced *FOXP3* (Treg‐associated) expression (*p* < 0.05, Figure 5A). MSC treatment significantly suppressed *RORγt* while enhancing *FOXP3* (*p* < 0.05), although *PU.1* showed a non‐significant downward trend (*p* > 0.05). Correlation analysis further revealed strong positive associations between AI and *RORγt* (*r* = 0.7847, *p* < 0.05) or *PU.1* (*r* = 0.6704, *p* < 0.05), whereas *FOXP3* correlated negatively with AI (*r* = −0.5335, *p* < 0.05) (Figure 5B–D). Western Blot analysis of these factors was consistent with RT‐qPCR results (Figure 5E–I). These findings suggest that dysregulated T lymphocyte differentiation contributes to CIA progression and that MSCs may mitigate joint injury by modulating this imbalance.

**Figure 6:**
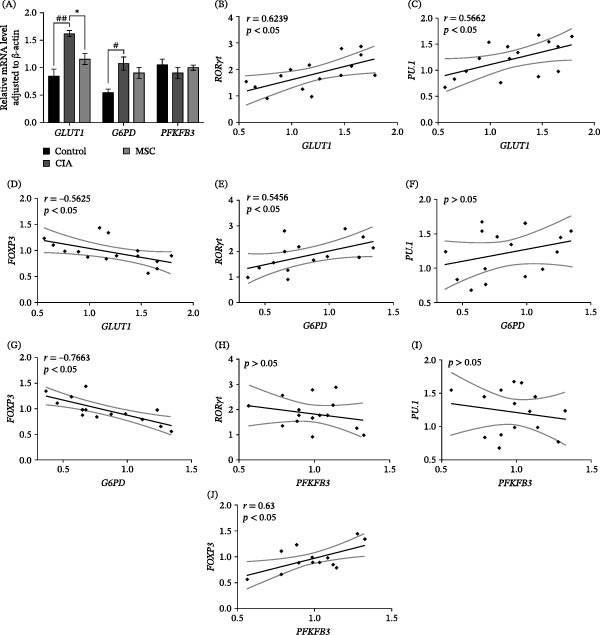
Effect of MSCs on the transcriptional levels of genes related to glucose metabolism in the spleen. (A) Relative mRNA levels of *GLUT1*, *G6PD*, and *PFKFB3*. (B) Correlations between *GLUT1* and *RORγt*. (C) Correlations between *GLUT1* and *PU.1*. (D) Correlations between *GLUT1* and *FOXP3*. (E) Correlations between *G6PD* and *RORγt*. (F) Correlations between *G6PD* and *PU.1*. (G) Correlations between *G6PD* and *FOXP3*. (H) Correlations between *PFKFB3* and *RORγt*. (I) Correlations between *PFKFB3* and *PU.1*. (J) Correlations between *PFKFB3* and *FOXP3*. Data are represent the mean ± SD (Control *n* = 5, CIA *n* = 5, and MSC *n* = 5). Data were analyzed via one‐way ANOVA followed by Tukey’s *post hoc* tests. Correlations were evaluated using Pearson or Spearman tests. ^#^ *p* < 0.05 Control vs. CIA, ^##^ *p* < 0.01 control vs. CIA, and  ^∗^ *p* < 0.05 CIA vs. MSC.

Quantitative analysis revealed significantly elevated *GLUT1* and *G6PD* mRNA levels in CIA mice compared to controls (*p* < 0.05). MSC treatment significantly downregulated *GLUT1* expression (*p* < 0.05) and showed a nonsignificant decreasing trend for *G6PD* (*p* > 0.05). Across all groups, *PFKFB3* mRNA expression remained unchanged without any notable difference (Figure 6A). Correlation analysis demonstrated significant positive associations between *GLUT1* mRNA and both *RORγt* (*r* = 0.6239, *p* < 0.05, Figure 6B) and *PU.1* mRNA (*r* = 0.5662, *p* < 0.05, Figure 6C), but a negative correlation with *FOXP3* mRNA (*r* = −0.5625, *p* < 0.05, Figure 6D). Similarly, *G6PD* mRNA showed positive correlated with *RORγt* mRNA (*r* = 0.5456, *p* < 0.05, Figure 6E) and negative correlation with *FOXP3* (*r* = −0.7663, *p* < 0.05, Figure 6G), but no significant association with *PU.1* (*p* > 0.05, Figure 6F). Interestingly, *PFKFB3* mRNA exhibited a positive correlation with*FOXP3* (*r* = 0.63, *p* < 0.05) (Figure 6J) but no significant associations with *RORγt* or *PU.1* (*p* > 0.05, Figure 6H,I). These findings suggest that MSCs may ameliorate RA‐associated joint inflammation by modulating glucose metabolism in T cells, potentially through suppressing *GLUT1*‐mediated metabolic pathways in pro‐inflammatory Th17/Th9 cells while promoting *PFKFB3*‐associated Treg cell function.

### 3.4. Alterations in Systemic Cytokine and Glycolytic Metabolism by MSCs

**Figure 7:**
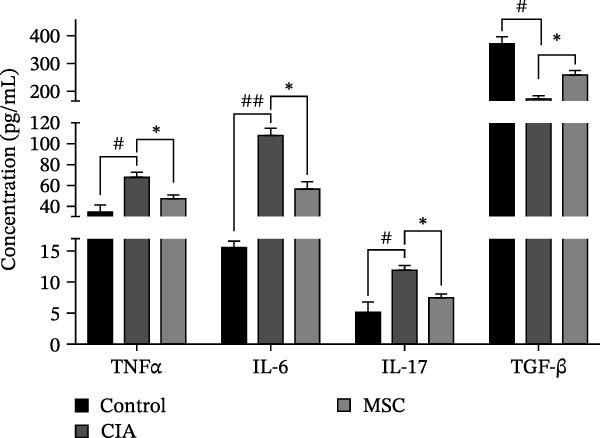
Effect of MSCs on cytokines. ELISA analysis of TNF‐α, IL‐6, IL‐17, and TGF‐β expression in serum after MSC intervention (control *n* = 4, CIA *n* = 9, and MSC *n* = 8). Data are presented as mean ± SD and were analyzed via one‐way ANOVA followed by Tukey’s *post hoc* tests. ^#^ *p* < 0.05 Control vs. CIA, ^##^ *p* < 0.01 control vs. CIA, and  ^∗^ *p* < 0.05 CIA vs. MSC.

CIA mice exhibited significantly elevated serum levels of pro‐inflammatory cytokines (TNF‐α, IL‐6, and IL‐17) compared to controls, which were markedly reduced following MSC treatment (both *p* < 0.05). Conversely, serum TGF‐β levels were significantly lower in CIA mice and restored by MSC administration (both *p* < 0.05) (Figure 7).

**Figure 8:**
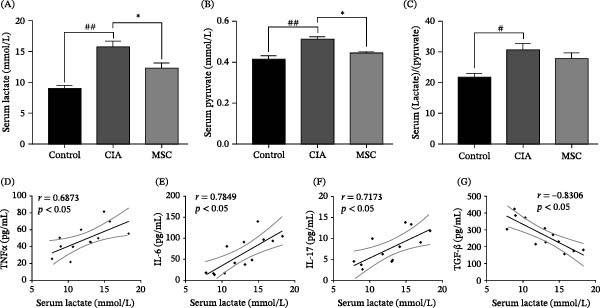
Effect of MSCs on serum lactate and pyruvate levels. (A) Serum lactate level. (B) Serum pyruvate level. (C) Serum lactate‐to‐pyruvate ratio. (D) Correlations between lactate and TNF‐α. (E) Correlations between lactate and IL‐6. (F) Correlations between lactate and IL‐17. (G) Correlations between lactate and TGF‐β. Data represents the mean ± SD from at least three independent experiments (Control *n* = 4, CIA *n* = 4, and MSC *n* = 5). Data were analyzed via one‐way ANOVA followed by Tukey’s *post hoc* tests. Correlations were evaluated using Pearson or Spearman tests. ^#^ *p* < 0.05 Control vs. CIA, ^##^ *p* < 0.01 control vs. CIA, and  ^∗^ *p* < 0.05 MSC vs. CIA.

Analysis revealed substantial increases in both serum lactate (15.86 ± 1.90 vs. 9.00 ± 1.04, *p* < 0.01) and pyruvate (0.52 ± 0.02 vs. 0.42 ± 0.03, *p* < 0.01) in CIA mice. MSC treatment partially normalized these levels (lactate: 12.39 ± 1.50, pyruvate: 0.45 ± 0.01, both *p* < 0.05 vs. CIA) (Figure 8A,B). The lactate‐to‐pyruvate ratio was significantly elevated in CIA mice (*p* < 0.05) and showed a nonsignificant decreasing trend after MSC treatment (*p* > 0.05, Figure 8C).

Notably, serum lactate levels strongly correlated with pro‐inflammatory cytokines (TNF‐α: *r* = 0.6873, IL‐6: *r* = 0.7849, IL‐17: *r* = 0.7173, all *p* < 0.05) and inversely with TGF‐β (*r* = −0.8306, *p* < 0.05) (Figure 8D–G). These findings indicate that MSC treatment ameliorates inflammatory and metabolic dysregulation in CIA mice by restoring cytokine balance and normalizing glycolytic metabolism, suggesting a potential mechanism through which MSCs exert therapeutic effects in RA.

### 3.5. Direct Modulation of Human CD4^+^ T Cell Differentiation by MSCs *In Vitro*

**Figure 9:**
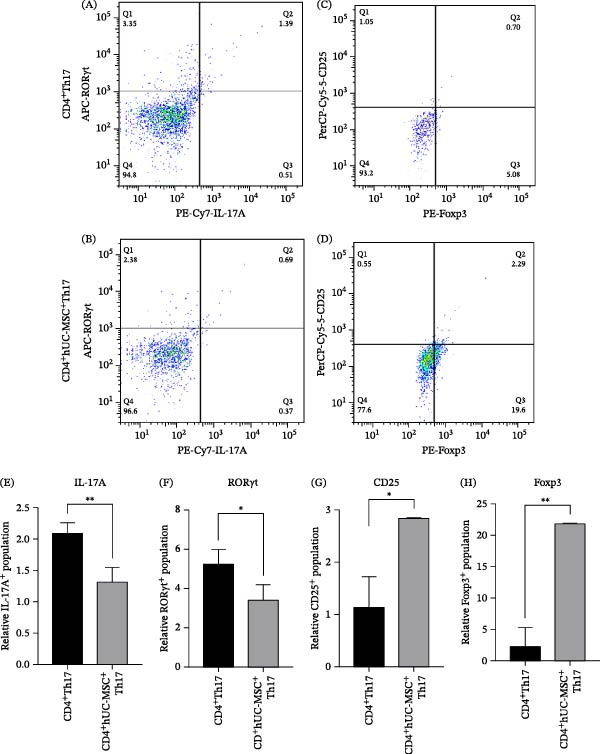
Effect of hUC‐MSCs on Th17/Treg differentiation in human CD4^+^ T cell *in vitro*. Primary human CD4^+^ T cells were gated for the staining of IL‐17A and *RORγt* representing Th17 cells under Th17‐polarizing conditions. Representative flow cytometric plots illustrating the frequencies of Th17 cells (CD4^+^IL‐17A^+^ or CD4^+^RORγt^+^, quadrant 2) in Th17‐polarizing conditions (A) and MSC coculture conditions (B). Representative flow cytometric plots illustrating the frequencies of Tregs (CD4^+^CD25^+^ or CD4^+^Foxp3^+^, quadrant 2) in Th17‐polarizing conditions (C) and MSC coculture conditions (D). Flow cytometric detection revealed downregulation of IL‐17A^+^ cells (E) and RORγt^+^ cells (F) in the CD4^+^hUC‐MSC^+^Th17 group, respectively. Flow cytometric detection revealed upregulation of CD25^+^ cells (G) and Foxp3^+^ cells (H) in the MSC treated group. The gating strategy is provided in Figures S5E and S10F 1–2. Results are representative of three independent biological replicates (*n* = 3 healthy donors). Data are presented as mean ± SD and were analyzed via unpaired two‐tailed Student’s *t*‐test.  ^∗^ *p* < 0.05;  ^∗∗^ *p* < 0.01 vs. Th17‐polarized control.

To directly assess the immunomodulatory effects of MSCs on human T cells, purified CD4^+^ T cells were cocultured with hUC‐MSCs under Th17‐polarizing conditions. Flow cytometric analysis revealed that MSCs significantly altered T cell differentiation (Figure 9A–D). Specifically, the proportion of Th17 cells (CD4^+^IL‐17A^+^ or CD4^+^RORγt^+^ cells) was substantially reduced upon MSC coculture (Figure 9E,F). In contrast, the frequency of Tregs (CD4^+^CD25^+^Foxp3^+^ cells) was markedly increased in the MSC coculture group compared to the Th17‐polarized control (Figure 9G,H). These results demonstrate that hUC‐MSCs directly promote Treg differentiation while suppressing Th17 polarization in human CD4^+^ T cells, supporting their immunoregulatory role in rebalancing the Th17/Treg axis.

### 3.6. Metabolic and Molecular Reprogramming in MSC Cocultured Human T Cells

**Figure 10:**
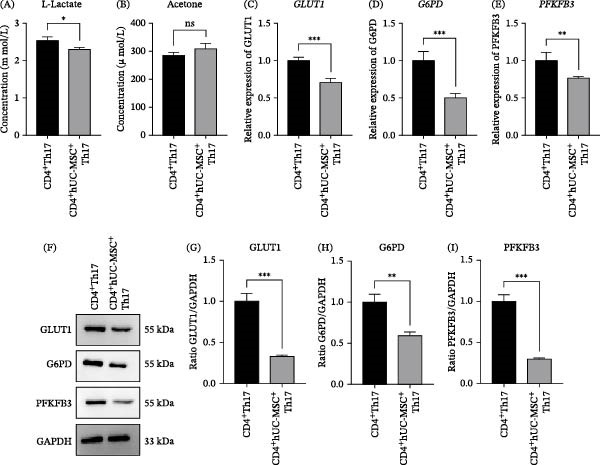
Effect of MSCs on metabolic and molecular reprogramming in human T Cells. Levels of L‐lactate (A) and acetone (B) in the culture supernatant were measured by colorimetric assay. mRNA expression of *GLUT1* (C), *G6PD* (D), and *PFKFB3* (E) was determined by RT‐qPCR and normalized to *GAPDH*. Representative western blot images (F) and quantitative analysis of GLUT1 (G), G6PD (H), and PFKFB3 (I) protein expression in CD4^+^ T cells, normalized to GAPDH. All uncropped raw western blot images were provided in the “Original Blot Data” PDF file. Data are presented as mean ± SD from three independent experiments. Statistical significance was calculated using an unpaired two‐tailed Student’s *t*‐test.  ^∗^ *p* < 0.05;  ^∗∗^ *p* < 0.01;  ^∗∗∗^ *p* < 0.001; ns, not significant vs. Th17‐polarized control.

To investigate whether hUC‐MSCs influence glucose metabolism in T cells, we analyzed metabolites in the culture supernatant and performed molecular profiling of the harvested T cells. Colorimetric assays revealed that L‐lactate levels in the supernatant were significantly lower in the hUC‐MSC coculture group compared to the Th17‐polarized control group (Figure 10A). In contrast, acetone levels showed a decreasing trend, though this did not reach statistical significance (Figure 10B). These findings suggest that hUC‐MSCs suppress glycolytic activity in T cells.

Consistent with the metabolic shift, RT‐qPCR analysis demonstrated that mRNA expression of key glycolytic genes, including *GLUT1*, *G6PD*, and *PFKFB3*, was significantly downregulated in T cells cocultured with hUC‐MSCs compared to controls (Figure 10C–E). Western blot analysis further confirmed these changes at the protein level. The expression of GLUT1, G6PD, and PFKFB3 proteins was markedly reduced in T cells from the hUC‐MSC coculture group (Figure 10F–I), aligning with the transcriptional and metabolic findings.

Collectively, these results indicate that hUC‐MSCs induce metabolic reprogramming in human CD4^+^ T cells, characterized by suppressed glycolysis and downregulation of key metabolic enzymes, which may contribute to the observed shift from pro‐inflammatory Th17 toward Treg cell differentiation.

## 4. Discussion

This study elucidates the therapeutic mechanisms of hUC‐MSCs in the CIA model. Our findings demonstrate that MSC administration mitigate joint damage and systemic inflammation not only by restoring the Th17/Treg balance but also through a novel mechanism involving the metabolic reprogramming of T cells. These results deepen our understanding of RA pathogenesis and underscore the potential of MSCs as a multitarget therapeutic strategy.

The critical role of glucose metabolism in RA progression is increasingly recognized. This is consistent with clinical observations in RA patients and previous preclinical studies [22]. CIA mice in our study exhibited a pronounced dysregulation in systemic glucose metabolism, characterized by elevated serum levels of lactate and pyruvate. This indicates a shift toward enhanced glycolytic activity, a metabolic state that fuels the activation and proliferation of inflammatory immune cells [23–25]. Notably, MSC treatment effectively normalized these metabolic parameters, suggesting a capacity to modulate systemic glucose flux. This aligns with emerging evidence that MSCs possess immunometabolic regulatory functions,capable of altering the metabolic landscape of immune cells [26, 27].

Our data provide a direct link between this metabolic modulation and T cell fate. *In vitro* coculture experiments demonstrated that hUC‐MSCs directly suppressed glycolytic activity in human CD4^+^ T cells, as evidenced by reduced lactate production and the downregulation of key glycolytic enzymes (*GLUT1*, *G6PD*, and *PFKFB3*) at both transcriptional and protein levels. This metabolic reprogramming was functionally coupled to a shift in T cell differentiation: a suppression of pro‐inflammatory Th17 cells and a promotion of Treg cells. The strong correlations observed *in vivo* between glycolytic markers (*GLUT1* and *G6PD*) and pro‐inflammatory transcription factors (*RORγt*, *PU.1*) and the inverse correlation with *FOXP3* further support the notion that MSCs restore immune homeostasis, at least in part, by diverting T cell metabolism away from glycolysis.

Concomitant with this metabolic and immunologic rebalancing, MSC treatment led to a significant reduction in pro‐inflammatory cytokines (TNF‐α, IL‐6, and IL‐17) and a restoration of anti‐inflammatory TGF‐β levels in the serum of CIA mice. The strong correlations between serum lactate levels and these cytokines underscore the interplay between metabolism and inflammation. By dampening the hyperactive immune response and correcting the cytokine imbalance, MSCs help restore homeostasis in the inflammatory microenvironment, contributing to the observed amelioration of joint pathology and clinical scores [18, 28].

The re‐establishment of the Th17/Treg cell equilibrium is a central event in MSC‐mediated therapy. Our results show that MSCs enhance the expression of the Treg master regulator FOXP3 while suppressing the Th17‐associated factor RORγt. This immunomodulatory effect is mechanistically linked to the aforementioned metabolic regulation as glycolysis is a known driver of Th17 differentiation, whereas Tregs often rely on oxidative phosphorylation [29, 30]. By modulating the metabolic substrate, MSCs create a microenvironment favorable for Treg expansion and suppressive function, offering a novel perspective on their therapeutic action.

Notably, this study has several limitations. First, the metabolic analysis was primarily conducted *in vitro* and on systemic (serum) levels *in vivo*. While the serum changes reflect systemic immunometabolic reprogramming, they may not fully represent the hypoxic and inflamed microenvironment within the arthritic joint. Future studies should directly measure metabolites and metabolic enzyme expression in synovial fluid or joint‐infiltrating T cells. Second, our investigation focused on a specific set of glycolytic markers. The involvement of other metabolic pathways, such as the pentose phosphate pathway or fatty acid oxidation, in MSC‐mediated immunomodulation remains undefined and warrants further exploration. Third, while the *in vitro* coculture system provides direct evidence, the sample size for human cell experiments was limited. Future research with larger cohorts and more comprehensive metabolic profiling is needed to fully delineate the immunometabolic network regulated by MSCs.

## 5. Conclusion

Our findings demonstrate that hUC‐MSCs alleviate CIA by orchestrating a dual immunomodulatory and metabolic reprogramming of T cells. They suppress pathogenic Th17 responses and promote Treg function, processes intimately linked to the inhibition of glycolysis. This study not only provides novel mechanistic insights into MSC‐based therapy for RA but also highlights the modulation of immunometabolism as a promising therapeutic avenue for autoimmune diseases.

## Author Contributions

Aijing Liu designed the study protocol and acted as the guarantor for the manuscript. Xiaoping Wang, Jingjing He, Qun Wang, and Xue Liu established the CIA mice model, conducted MSCs treatment in arthritis mice, and drafted the manuscript. Haoming Yuan, Lu Jin, Meng Ding, Lin Yang, and Shaoxin Cui conducted pathological staining and detection of metabolic products and genes. Fei Chang, Tong Xin, and Hongtao Jin conducted cytokines detection and statistical analysis and contributed to the drafting of the review. Aijing Liu, Wensen Pan, Yongzhou Song, and Min Shi critically revised the manuscript for important intellectual content.

## Funding

This work was supported by the Medical Science Research Project of Hebei (Grant 20240127), the Natural Science Foundation of Hebei Province (Grant H2023206264), and the Government Foundation of Excellent Clinical Medicine Talent Program of Hebei (Grant ZF2025100).

## Disclosure

All authors approved the final version of the article. The corresponding author attests that all listed authors meet the authorship criteria and that no others meeting the criteria have been omitted.

## Ethics Statement

The experimental procedures were conducted in accordance with guidelines approved by the Animal Ethics Committee of The Second Hospital of Hebei Medical University (Approval Number: 2022‐AE292).

## Conflicts of Interest

The authors declare no conflicts of interest.

## Supporting Information

Additional supporting information can be found online in the Supporting Information section.

## Supporting information


**Supporting Information** File S1: The supporting data for this article consists of one PDF file titled “Original Blot Data.pdf”. This file contains the full‐length, uncropped western blot images corresponding to the representative protein expression data presented in Figures S5 and S10. All membranes were processed and imaged under consistent experimental conditions as described in the Materials and Methods section.

## Data Availability

The data that support the findings of this study are available from the corresponding author upon reasonable request.
