# Supplementary material for: Metabolic Reprogramming of T Cells by MSCs Rebalances Th17/Treg Axis to Attenuate Collagen‐Induced Arthritis
Source: J Immunol Res. 2026 Jun 8;2026:1862250. doi: 10.1155/jimr/1862250 (PMC13247138; doi:10.1155/jimr/1862250)
Supplement: Supplementary file 1 — Supporting Information File S1: The supporting data for this article consists of one PDF file titled “Original Blot Data.pdf”. This file contains the full‐length, uncropped western blot images corresponding to the representative protein expression data presented in Figures S5 and S10. All membranes were processed and imaged under consistent experimental conditions as described in the Materials and Methods section. [file JIMR-2026-1862250-s001.pdf]

The supplementary data for this article consists of one PDF file titled "Original Blot Data.pdf". This file contains the full-length, uncropped western blot images corresponding to the representative protein expression data presented in Figure 5 and Figure 10. All membranes were processed and imaged under consistent experimental conditions as described in the Materials and Methods section.

Figure 5E

RORγt

β-actin

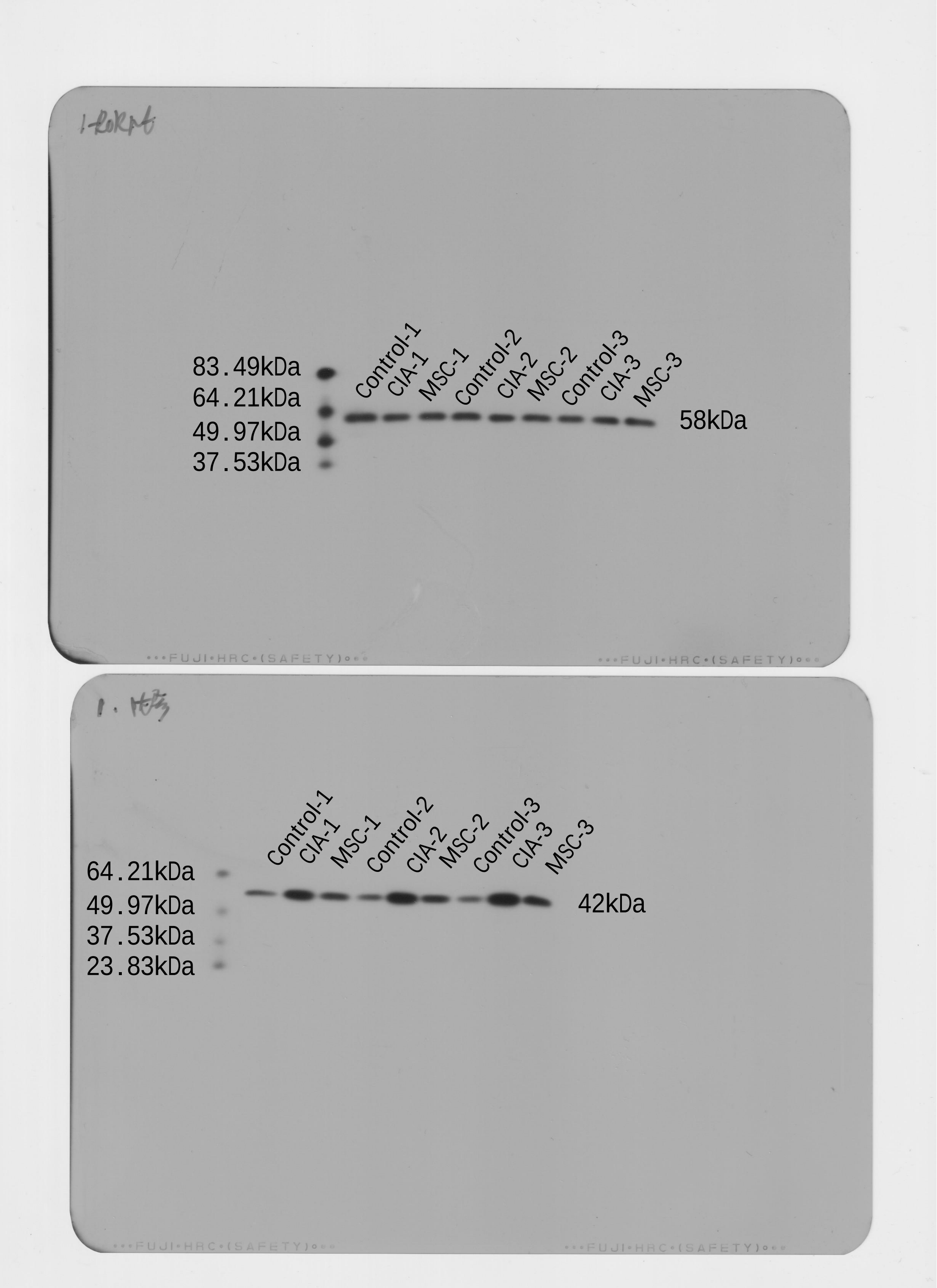

β -actin

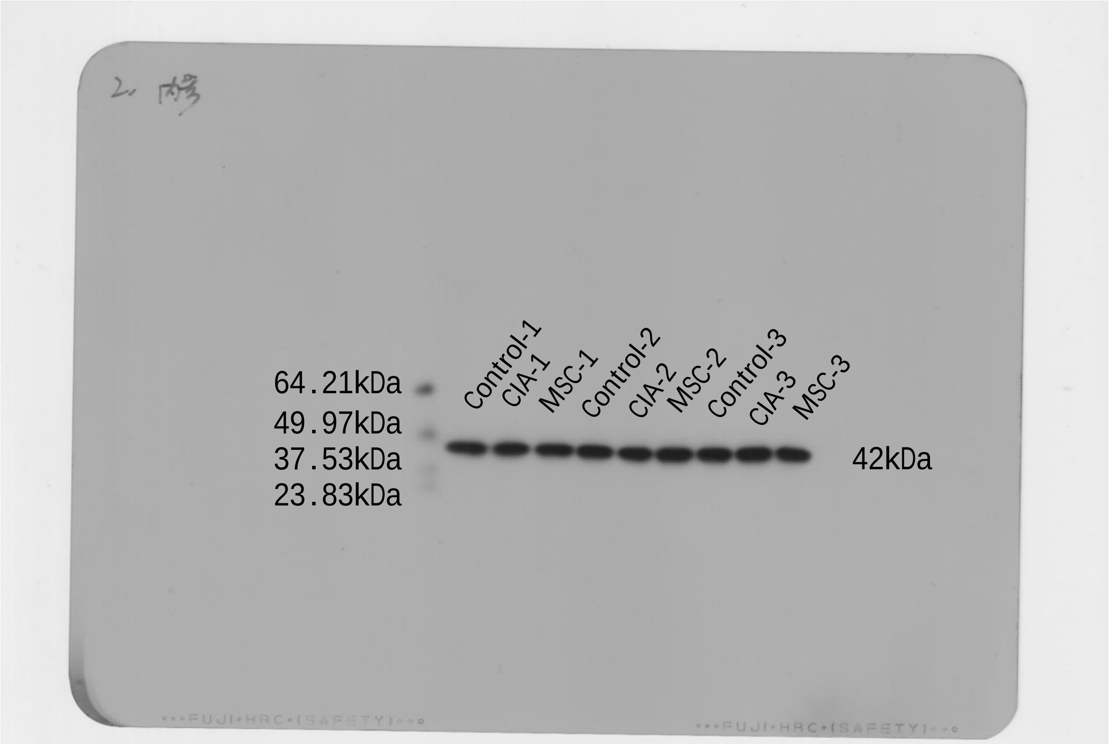

FOXP3

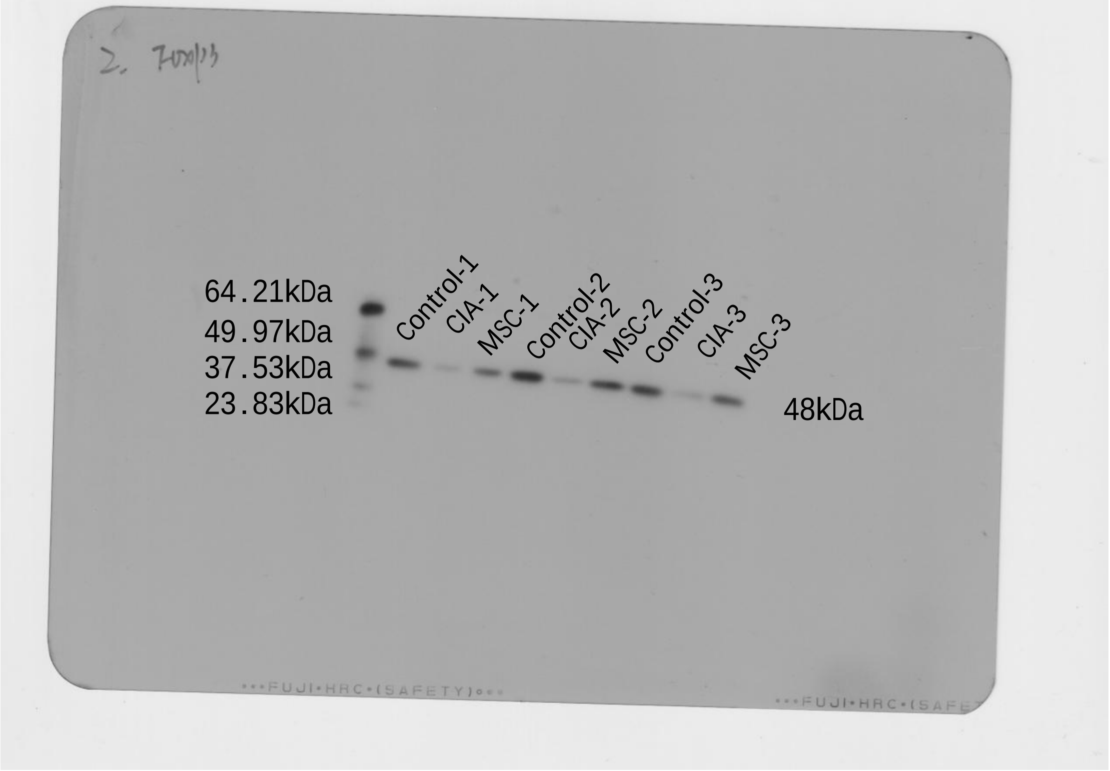

β -actin

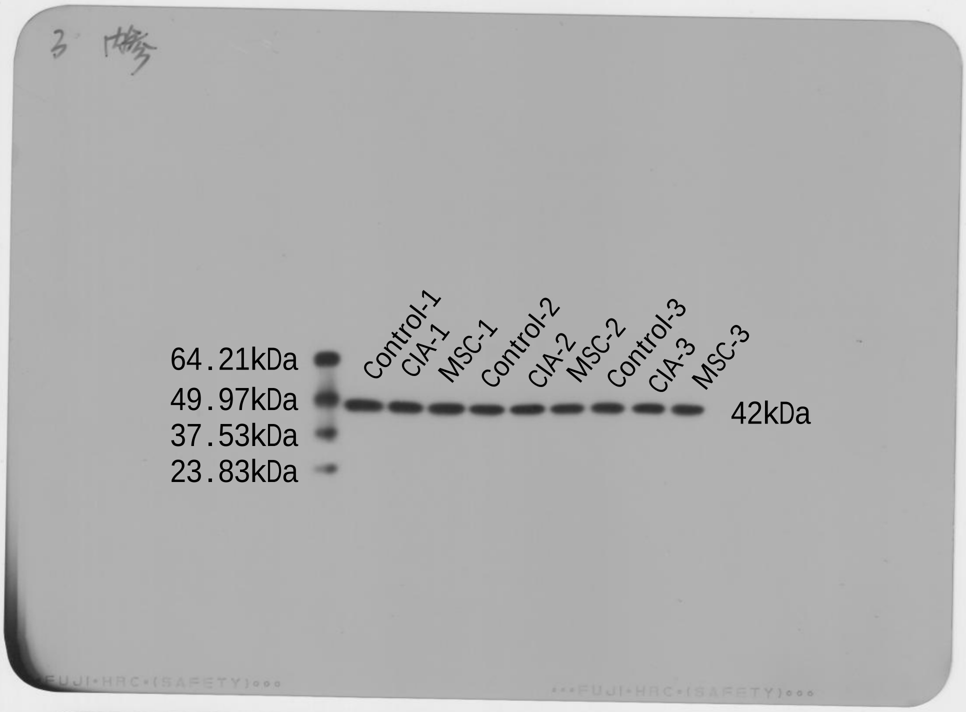
64.21kDa
49.97kDa
37.53kDa
23.83kDa
Control-1
CIA-1
MSC-1
Control-2
CIA-2
MSC-2
Control-3
CIA-3
MSC-3
42kDa

PU.1

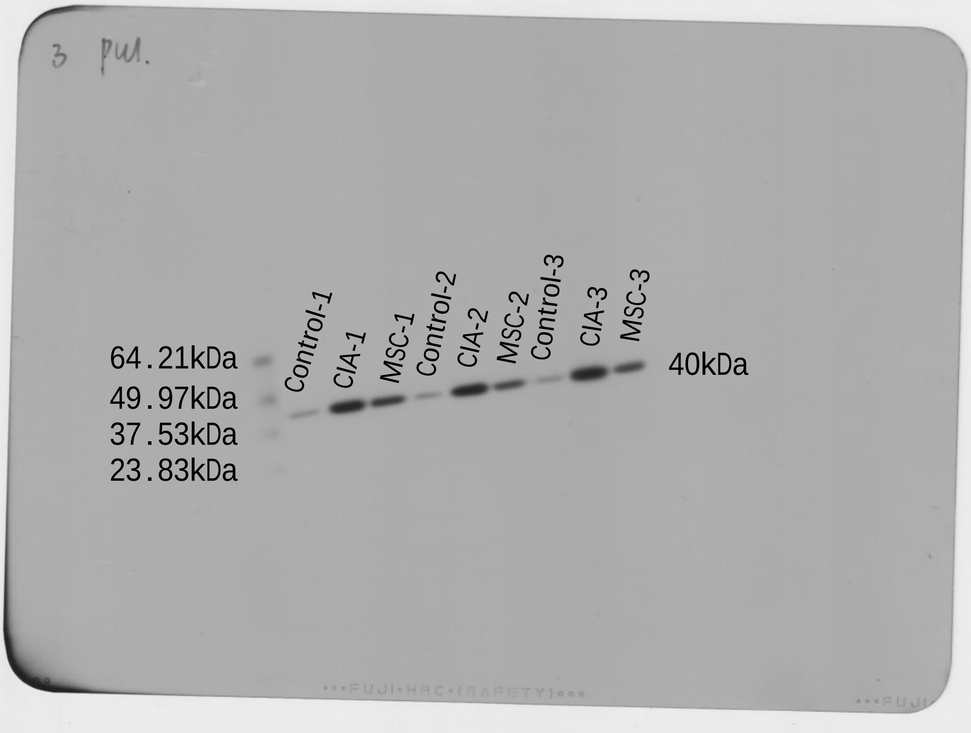
64.21kDa
49.97kDa
37.53kDa
23.83kDa
Control-1
CIA-1
MSC-1
Control-2
CIA-2
MSC-2
Control-3
CIA-3
MSC-3
40kDa

β -actin

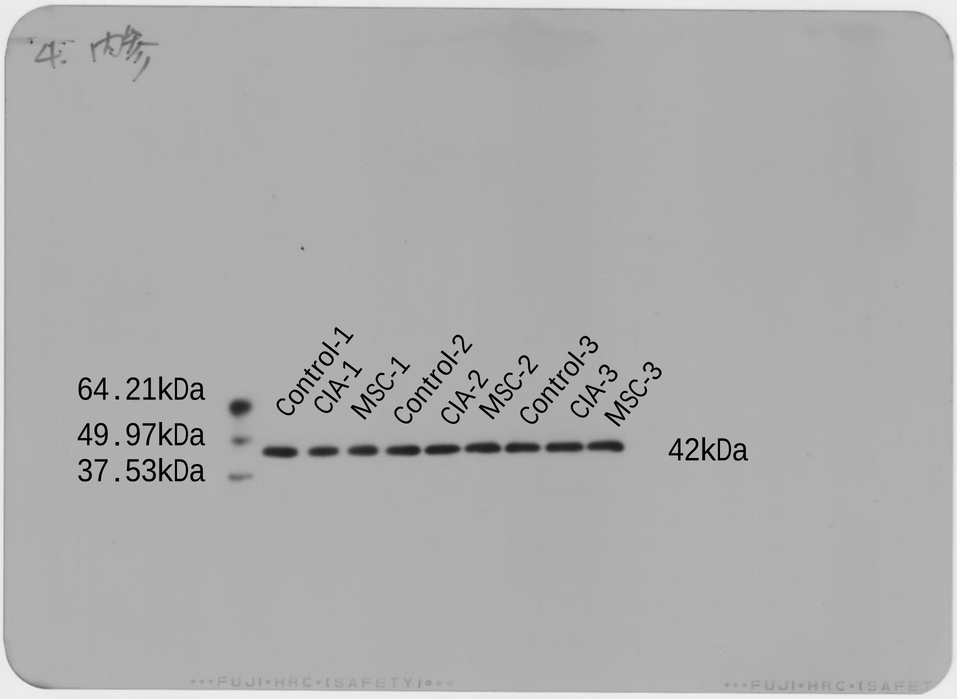
Control-1
CIA-1
MSC-1
Control-2
CIA-2
MSC-2
Control-3
CIA-3
MSC-3
64.21kDa
49.97kDa
37.53kDa
42kDa

IL-9

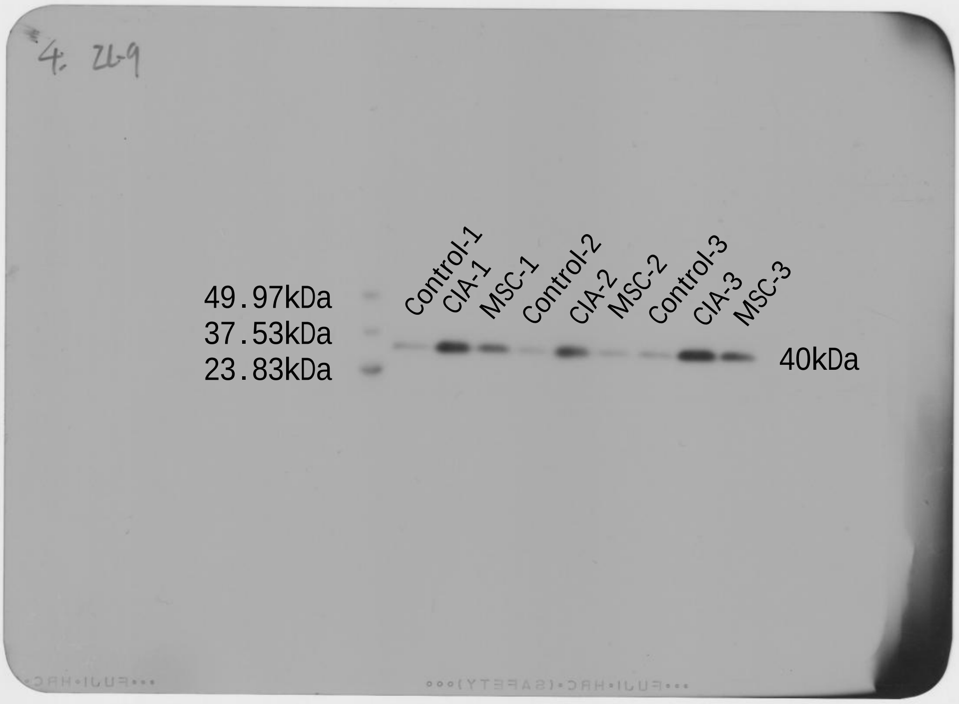
Control-1
CIA-1
MSC-1
Control-2
CIA-2
MSC-2
Control-3
CIA-3
MSC-3
49.97kDa
37.53kDa
23.83kDa
40kDa

Figure 10F

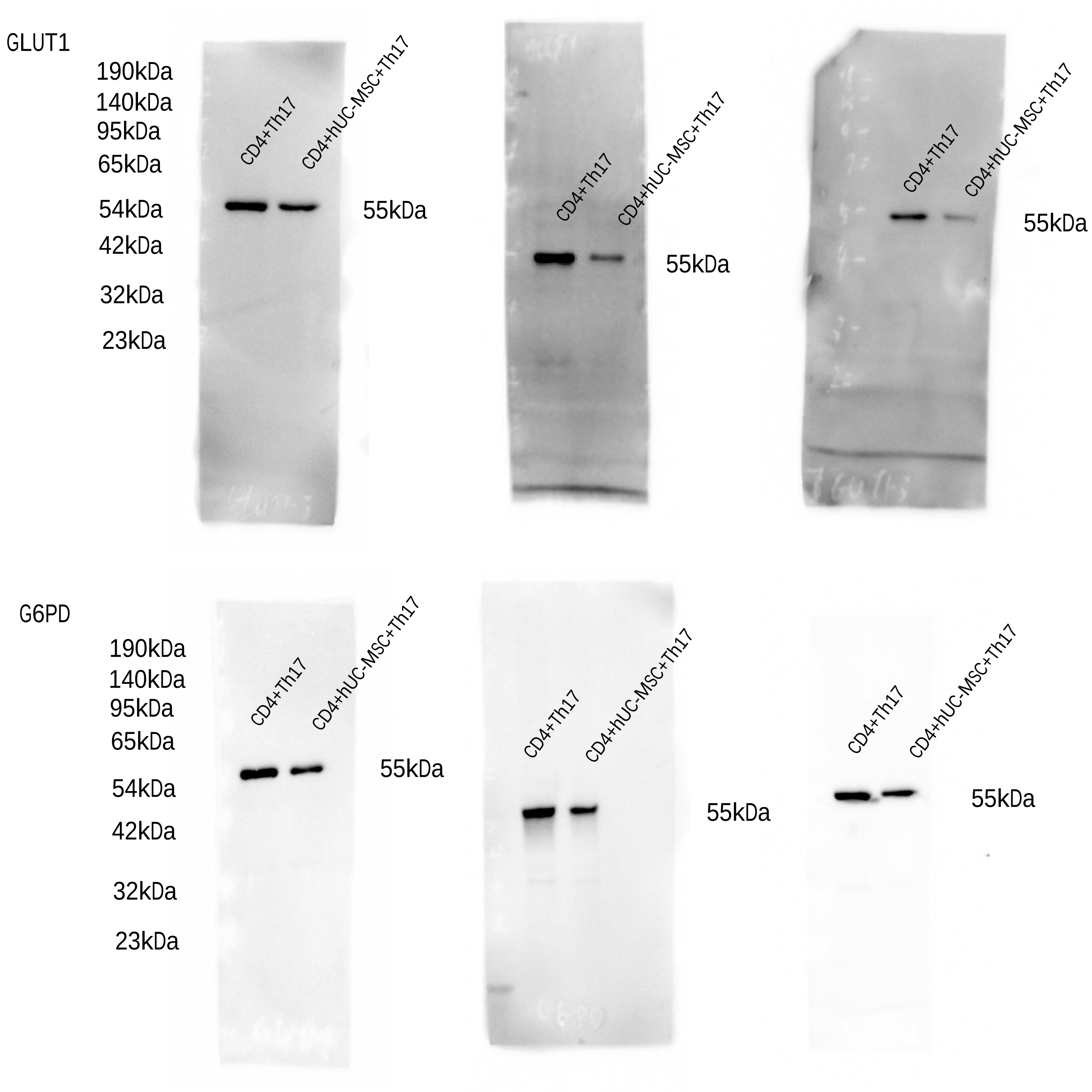
GLUT1
190kDa
140kDa
95kDa
65kDa
54kDa
42kDa
32kDa
23kDa
CD4+Th17
CD4+hUC-MSC+Th17
55kDa
CD4+Th17
CD4+hUC-MSC+Th17
55kDa
CD4+Th17
CD4+hUC-MSC+Th17
55kDa
G6PD
190kDa
140kDa
95kDa
65kDa
54kDa
42kDa
32kDa
23kDa
CD4+Th17
CD4+hUC-MSC+Th17
55kDa
CD4+Th17
CD4+hUC-MSC+Th17
55kDa
CD4+Th17
CD4+hUC-MSC+Th17
55kDa

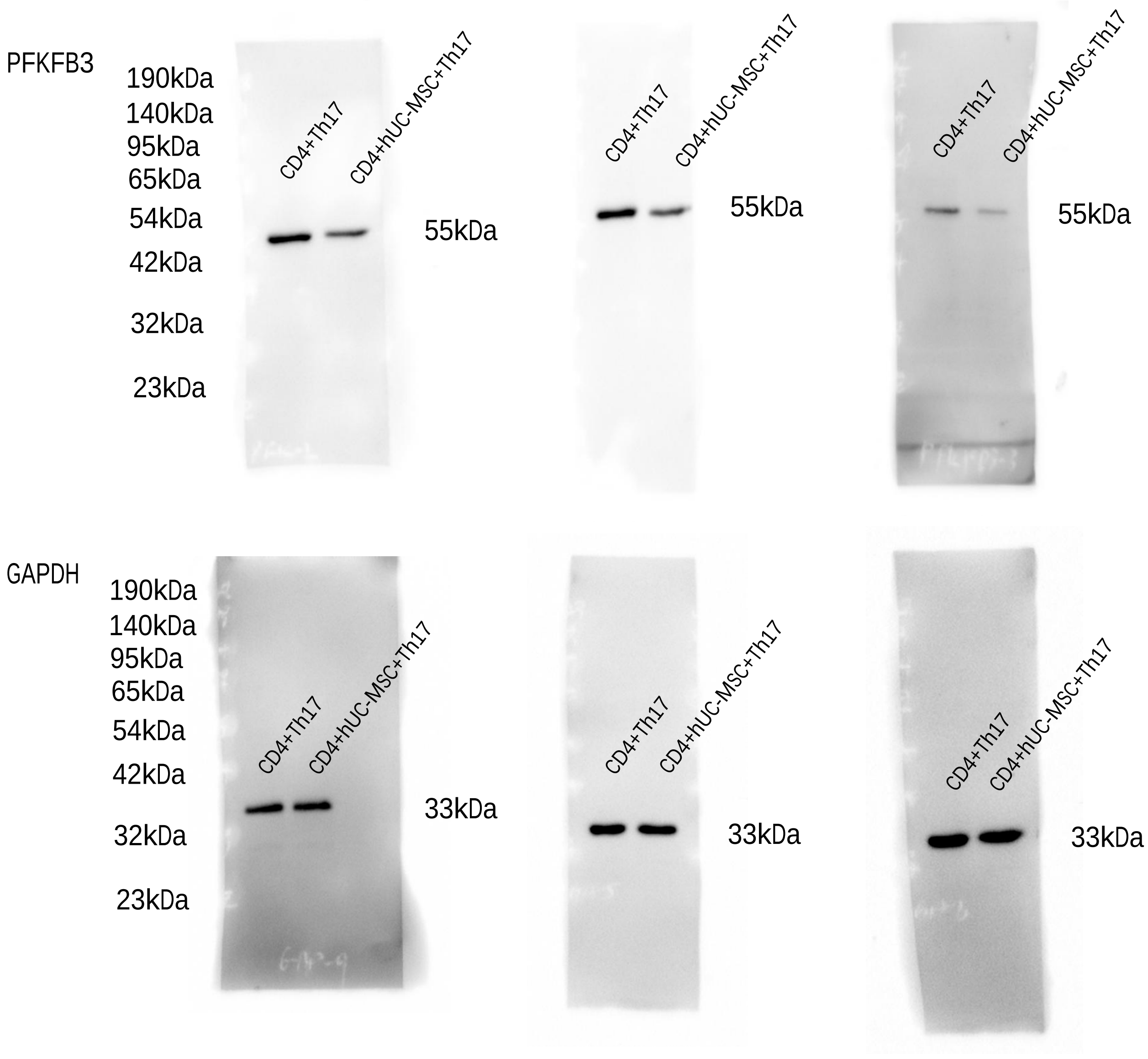
PFKFB3
190kDa
140kDa
95kDa
65kDa
54kDa
42kDa
32kDa
23kDa
CD4+Th17
CD4+hUC-MSC+Th17
55kDa
CD4+Th17
CD4+hUC-MSC+Th17
55kDa
CD4+Th17
CD4+hUC-MSC+Th17
55kDa
GAPDH
190kDa
140kDa
95kDa
65kDa
54kDa
42kDa
32kDa
23kDa
CD4+Th17
CD4+hUC-MSC+Th17
33kDa
CD4+Th17
CD4+hUC-MSC+Th17
33kDa
CD4+Th17
CD4+hUC-MSC+Th17
33kDa
